# MaSter-Bio – Messinstrument für das akademische Selbstkonzept zum technologiebezogenen Professionswissen von angehenden Biologielehrpersonen

**DOI:** 10.1007/s40573-022-00137-6

**Published:** 2022-04-05

**Authors:** Daniela Mahler, Julia Arnold

**Affiliations:** 1 Didaktik der Biologie, Freie Universität Berlin, Schwendenerstr. 1, 14195 Berlin, Deutschland; 2 Fachhochschule Nordwestschweiz FHNW – Pädagogische Hochschule, ZNTD – Zentrum für Naturwissenschafts- und Technikdidaktik, Hofackerstraße 30, 4132 Muttenz, Schweiz

**Keywords:** Akademisches Selbstkonzept, TPACK, Validierung, Fragebogen, Biologielehrkräfte, Digitale Technologien, Academic self-concept, TPACK, Validation, Questionnaire, TPACK, Biology teacher

## Abstract

Digitale Technologien können – sinnvoll eingesetzt – das Lernen befördern. Ob jedoch die Potenziale digitaler Technologien im Regelunterricht tatsächlich genutzt werden, hängt zu einem relevanten Maß von der Lehrperson und ihrer Bereitschaft zur Nutzung ab. Hierbei ist nicht zuletzt das akademische Selbstkonzept der Lehrpersonen in Bezug auf das technologiebezogene Professionswissen (*technological pedagogical content knowledge* – TPACK) von Bedeutung. Um dieses Konstrukt in seiner Tiefe zu verstehen oder den Erfolg von Förderangeboten abschätzen zu können, ist die valide und reliable Messung des akademischen Selbstkonzeptes zum technologiebezogenen Professionswissen wichtig. In diesem Beitrag wird der MaSter-Bio als ein Messinstrument für das akademische Selbstkonzept zum technologiebezogenen Professionswissen von angehenden Biologielehrpersonen vorgestellt. Bei seiner Entwicklung wurden die aktuelle Forschung zum akademischen Selbstkonzept und zum technologiebezogenen Professionswissen, die Nutzbarkeit im deutschsprachigen Raum sowie eine spezifische Ausschärfung für den Biologieunterricht berücksichtigt. Reliabilität und Validität wurden an einer Stichprobe von 403 angehenden Biologielehrpersonen überprüft. Mit dem Instrument können die sieben angenommenen Subskalen des Konstrukts reliabel erfasst werden und es liegen Hinweise für diskriminante und konvergente Validität vor.

## Abstract (en)

Digital technologies can—if meaningfully integrated into the lessons—promote learning. However, whether the potentials of digital technologies are actually used in the regular classroom depends to a relevant degree on the teacher and his or her willingness to do so. One important factor for this willingness is the teachers’ academic self-concept related to technological pedagogical content knowledge (TPACK). In order to understand the construct in its depth and to be able to assess the success of interventions in teacher education, the valid and reliable measurement of the academic self-concept related to technological pedagogical content knowledge is important. In this article, the MaSter-Bio is presented as a measurement instrument for the academic self-concept related to technological pedagogical content knowledge of prospective biology teachers. In its development, the current research on academic self-concept and technological pedagogical content knowledge, the usability in German-speaking countries, and a specific refinement for biology teaching were taken into account. Reliability and validity were tested on a sample of 403 prospective biology teachers. The instrument reliably measures the seven hypothesized subscales of the construct and provides sufficient evidence for discriminant and convergent validity.

## Einleitung

Schüler*innen der Sekundarstufe I dokumentieren gemeinsam Pflanzenarten, die sie auf dem Schulgelände finden und nutzen ihre Smartphones und Tablets, um die Funde fotografisch festzuhalten und mit Apps zu bestimmen. Anschließend laden sie die Fotos in die Schul-Cloud, um die Bestimmung und Sammlung zu dokumentieren. Zusätzlich stellen sie ihre Funde der Gemeinschaft (im Sinne von *citizen science*) zur Verfügung, indem sie diese auf einer kollaborativen Plattform hochladen.

Dieses Beispiel veranschaulicht, wie der Einsatz digitaler Technologien den Biologieunterricht bereichern kann, indem er den Erwerb von Artenkenntnis (Groß 2018) sowie das Teilen der Resultate mit der (wissenschaftlichen) Gemeinschaft erleichtert. Zusätzlich widerspricht dieses Beispiel der Annahme, dass digitale Technologien im Unterricht Primärerfahrungen eher verhindern oder ersetzen würden. Es zeigt aber auch, dass die Biologielehrperson ein geeignetes Lernsetting schaffen muss, um kognitive Passivität auf Schüler*innenseite beim Bestimmen durch die App zu vermeiden.

Unter *digitalen Technologien* fasst dieser Beitrag folgende Kategorien zusammen: Hardware (z. B. Tablets, Smartphones), Software (z. B. Programme zur Textverarbeitung oder Tabellenkalkulation, Lernsoftware), digitale Lernmedien (z. B. E‑Books, Videoinstruktion) sowie entsprechendes Zubehör (z. B. digitale Messgeräte). Solche digitalen Technologien können – sinnvoll eingesetzt – das Lernen allgemein (Tamin et al. 2011), den naturwissenschaftlichen Unterricht (Schmid et al. 2021) sowie den Biologieunterricht bereichern (Remmele und Schaal 2018; Schaal und Crossley, 2013). Um nur wenige Beispiele zu nennen: Apps zur Pflanzenbestimmung fördern – bei richtiger Einbettung in den Biologieunterricht – Arten- und Formenkenntnisse (Groß 2018), Erklärvideos können das Erlernen von Laborprozeduren unterstützen (Jordan et al. 2016; Nadelson et al. 2015) und Augmented Reality kann den Lernerfolg beim Lernen der menschlichen Anatomie erhöhen (Fokides 2018). Darüber hinaus können fachliche Inhalte mit Hilfe digitaler Technologien vertieft erlernt werden (Kramer et al. 2019). Viele Phänomene, die mit dem bloßen Auge nicht erfasst werden können, können mit digitalen Technologien sichtbar werden (Weitzel 2013). So können sich Lernende durch dynamische Repräsentationen wie Erklärvideos komplexe oder abstrakte Inhalte erschließen (Evagorou et al. 2015) oder die räumlichen Dimensionen eines (anatomischen) Gegenstandes besser verstehen (Ainsworth et al. 2010; Remmele et al. 2018). Zudem sind digitale Concept Maps eine gute Möglichkeit komplexe Zusammenhänge, wie sie bspw. in Ökosystemen auftreten, zu visualisieren (Weitzel 2013) und so den Aufbau konzeptuellen Wissens zu unterstützen. Ob jedoch die Potenziale digitaler Technologien im Regelunterricht tatsächlich genutzt werden, hängt zu einem relevanten Maß von der Lehrperson und ihrer Bereitschaft ab (Mahler und Arnold 2017; Petko et al. 2018). Die Kompetenzaspekte auf Lehrpersonenseite, die für den gewinnbringenden Einsatz digitaler Technologien im Unterricht eine Rolle spielen lassen sich unter dem Begriff professionelle Handlungskompetenz zusammenfassen (Baumert und Kunter 2006). Hierbei ist erstens das technologiebezogene Professionswissen einer Lehrperson (*technological pedagogical content knowledge* – TPACK; Mishra und Koehler 2006) – also jenes Wissen auf Lehrpersonenseite, welches für den lernförderlichen Einsatz digitaler Technologien notwendig ist – von Bedeutung (s. S. 6 ff. für eine ausführliche Beschreibung des Konstrukts). Motivationale Orientierungen beeinflussen zweitens, ob und in welchem Ausmaß dieses Wissen auch tatsächlich in Handeln mündet (Ajzen 2002; Mahler und Arnold 2017; Pintrich, 2003; Scherer und Teo 2019), da sie im Zusammenhang mit der Aufrechterhaltung von Absichten und der Regulation des beruflichen Verhaltens (Baumert und Kunter 2006) stehen. In diesem Zusammenhang steht insbesondere das akademische Selbstkonzept bezüglich des TPACK im Fokus zahlreicher Studien (z. B. Schmidt et al. 2009; s. S. 8).

Auffällig bei bestehenden Messinstrumenten ist jedoch, dass viele Autoren angeben, technologiebezogenes *Professionswissen* zu messen, dann aber ein Instrument zur Erfassung des akademischen *Selbstkonzepts* vorstellen (Pamuk et al. 2015; Schmidt et al. 2009; Tan et al. 2019; Valtonen et al. 2017). Wenn man allerdings davon ausgeht, eigentlich Wissen zu messen, ist die Entwicklung des Instrumentes nicht auf das eigentliche Konstrukt des Selbstkonzeptes ausgelegt. Selbstkonzept und das entsprechende Professionswissen korrelieren zwar moderat (z. B. Paulick et al. 2016), letztlich handelt es sich aber um zwei unterschiedliche Konstrukte, die auch im Modell professioneller Kompetenz (Baumert und Kunter 2006) unterschiedlichen Kompetenzaspekten zugeordnet werden (Professionswissen vs. motivationale Orientierungen). Entsprechend sind auch unterschiedliche Messinstrumente je nach Forschungsfrage zu wählen.

Instrumente zum TPACK-Selbstkonzept sind also bereits entwickelt worden (s. oben). Diese bieten einen sehr guten Ausgangspunkt zur Messung bei angehenden Biologielehrpersonen, haben allerdings – neben der oben beschriebenen Unschärfe des zu messenden Konstrukts – Defizite. Zunächst muss das Instrument zur Nutzung im deutschsprachigen Raum sprachlich angepasst werden. Darüber hinaus beziehen vorhandene Instrumente sich nicht auf ein spezifisches Fach, weshalb die Passung speziell für den *Biologieunterricht* nicht gegeben ist, obwohl sich gerade für die Forschung zur Handlungsrelevanz von Wissen gezeigt hat, dass die Passung zu den Inhalten möglichst nah sein sollte (*law of specifity*, Ajzen und Fishbein 2005). Es ist also davon auszugehen, je passgenauer die Situation, für die das Selbstkonzept abgefragt wird, zu der Situation ist, für die dann auch die Handlung untersucht wird, desto prädiktiver werden die Ergebnisse. Da es das Ziel ist, auf die Nutzung digitaler Technologien im Biologieunterricht zu schauen, ist die Fokussierung auf den Biologieunterricht hier sinnvoll.

Ziel des vorgestellten Projektes ist es, diese Aspekte zu berücksichtigen und ein reliables Instrument für den deutschsprachigen Raum zur validen Erfassung des akademischen TPACK-Selbstkonzeptes zu entwickeln. Da die Lehrer*innenbildung an Hochschulen eine höchst relevante Phase für die Professionalisierung von Lehrpersonen (Kunter et al. 2013a) und damit auch für die Entwicklung des akademischen Selbstkonzepts zum TPACK darstellt, fokussiert das vorgestellte Instrument Lehramtsstudierende sowie Lehrpersonen im Vorbereitungsdienst. Dabei liegt der Schwerpunkt dieses Beitrags auf folgenden Aspekten: (1) Berücksichtigung der Forschung zum akademischen Selbstkonzept und zum technologiebezogenen Professionswissen, (2) Nutzbarkeit im deutschsprachigen Raum, (3) eine spezifische Ausschärfung für den Biologieunterricht.

## Theoretischer Hintergrund

Verschiedene Studien zeigen, welche Potenziale digitale Technologien für den Biologieunterricht bieten (z. B. Bruckermann und Mahler 2019; Kramer et al. 2019). Diese Potenziale können sich allerdings nur entfalten, wenn digitale Technologien von der Biologielehrperson auch *tatsächlich* genutzt und in einen lernförderlichen Kontext eingebettet werden. Die Brücke zwischen der digitalen Technologie und dem Lernerfolg der Schüler*innen ist die Lehrperson mit ihrer professionellen Handlungskompetenz. Der folgende Abschnitt soll einen detaillierten Einblick in die berücksichtigten Konstrukte geben und diese im Modell professioneller Handlungskompetenz verorten. Wenn sich das akademische Selbstkonzept auf das Professionswissen einer Lehrperson bezieht, ist es für die Messung von größter Wichtigkeit, die zugrundeliegende Wissensbasis im Detail zu verstehen. Aus diesem Grund folgt ein Exkurs zum technologiebezogenen Professionswissen von Lehrpersonen, bevor Bezug auf das korrespondierende Selbstkonzept genommen wird.

### Professionelle Handlungskompetenz als Grundlage für den lernförderlichen Einsatz digitaler Technologien

Erfolgreiches Unterrichten erfordert unterschiedliche Eigenschaften auf Lehrpersonenseite, die sich im Modell professioneller Handlungskompetenz von Baumert und Kunter (2006) zusammenfassen lassen. Das Modell professioneller Handlungskompetenz ist ursprünglich nicht für den Kontext digitaler Technologien entwickelt worden, lädt jedoch aufgrund seines heuristischen Charakters explizit zu einer Ausdifferenzierung ein (Baumert und Kunter 2006).

Zahlreiche Studien widmeten sich bereits dem Professionswissen als Wissensbasis für das Unterrichten (z. B. Baumert et al. 2010; Großschedl et al. 2015). Diese Wissensbasis umfasst drei Bereiche, die sich entweder auf ein Fach beziehen oder fachunabhängig sind. Das fachbezogene Professionswissen vereint zwei Bereiche: das Wissen über fachbezogene Fakten sowie die Strukturen und Konzepte eines bestimmten Faches stellen die Facetten *des Fachwissens *dar (im Modell: *content knowledge – CK*). Das fachdidaktische Wissen (im Modell: *pedagogical content knowledge – PCK*) hilft Lehrpersonen ihren Schüler*innen fachliche Inhalte zu vermitteln (Shulman 1986). Vor allem zwei Facetten werden in der Literatur beschrieben (1) das Wissen über Repräsentationsformen und Instruktionsstrategien sowie (2) das Wissen über Schüler*innenkognitionen (z. B. fachspezifische Lernschwierigkeiten oder Schüler*innenvorstellungen; Großschedl et al. 2015; Schmelzing et al. 2013). Das pädagogische Wissen (im Modell: *pedagogical knowledge – PK*) ist das Wissen über das Lehren und Lernen (z. B. das Wissen über Klassenführung oder das Wissen über Unterrichtsmethoden unabhängig von einem bestimmten Fach; Baumert und Kunter 2006; Kleickmann et al. 2014). Eine technologiespezifische Weiterentwicklung der Überlegungen von Shulman (1986) nehmen Mishra und Koehler (2006) in ihrem TPACK-Modell vor. Dieses Modell ist deshalb vielversprechend, weil es den Einsatz von digitalen Technologien im Unterricht nicht isoliert betrachtet, sondern im Modell (a) oben genannte Wissensbereiche, die für jeden Unterricht relevant sind, (b) eine fachliche Perspektive sowie (c) spezifisch technologiebezogene Wissensbereiche vereint. Dabei vermeidet das TPACK-Modell digitale Technologien – das ist eine berechtigte Kritik, die auch heute noch immer wieder zum Technologieeinsatz im Unterricht aufkommt – um ihrer selbst willen zu nutzen. Weiterhin umgeht diese ganzheitliche Sichtweise einen Schwerpunkt auf reine technische Fähigkeiten oder „Bediener-Wissen“ (Graham 2011), die nicht in Zusammenhang mit dem Unterricht oder dem Fach stehen und entsprechend auch nicht zum Gelingen des Fachunterrichts beitragen können. Die Wissensbereiche im Modell haben immer einen oder mehrere Bezüge zu den Aspekten (1) Technologie, (2) Fach und (3) Unterricht (siehe Tab. 1).

**Table 1:** 

Wissensbereiche	Fachbezug	Unterrichtsbezug	Technologiebezug
PK	–	X	–
CK	X	–	–
PCK	X	X	–
TK	–	–	X
TPK	–	X	X
TCK	X	–	X
TPCK	X	X	X

Zu den oben beschriebenen technologieunabhängigen Wissensbereichen kommen bei Mishra und Koehler (2006) jene Wissensbereiche, die sich durch einen Technologiebezug auszeichnen. Diese Wissensbereiche sind konzeptuell mit den „klassischen“ Wissensbereichen verknüpft. Bezugnehmend auf Mishra und Koehler (2006), auf die die kommenden Ausführungen rekurrieren, stellt das Technologiewissen (im Modell: *technological knowledge – TK*) das Wissen über die digitalen Technologien selbst dar (für eine deutsche Übersichtsbeschreibung s. auch Mahler und Arnold 2017). Dieser Wissensbereich hat damit einen Technologiebezug, ist aber losgelöst vom Unterricht selbst und dem fachlichen Kontext zu verstehen. Zum einen geht es hier darum, wie man Hardware oder Software, z. B. eine App installiert. Zusätzlich sollten Lehrpersonen über eine Art Metawissen verfügen, beispielsweise dazu, wie Software entwickelt wird. Das technologiebezogene Fachwissen (im Modell: *technological content knowledge – TCK*) hat einen Technologie- sowie Fachbezug, jedoch keinen Bezug zum Unterricht. Das TCK bezieht sich auf das Wissen über Technologien im Fach wie beispielsweise digitale Messtechnik oder Software zur Datenverarbeitung und -visualisierung. Das technologiebezogene pädagogische Wissen (im Modell: *technological pedagogical knowledge –* TPK) zeichnet sich durch einen Technologie- sowie Unterrichtsbezug aus und bezeichnet das Wissen, das notwendig ist, um Technologien lernförderlich im Unterricht einzusetzen. Das TPK ist fachunabhängig. So umfasst es beispielsweise das Wissen darüber, wie Technologien sich auf das Lernklima auswirken oder wie Technologien allgemein zur Unterstützung des kollaborativen Arbeitens genutzt werden können. Der Wissensbereich, der alle Aspekte vereinigt – Technologien, Fach und Unterricht – ist das technologiebezogene fachdidaktische Wissen (im Modell: *technological pedagogical content knowledge – TPCK*), also das Wissen darüber, wie Technologien das Lernen im Fach unterstützen können. So umfasst dies beispielsweise das Wissen darüber, wie Simulationen das Wissen über Räuber- und Beute-Beziehungen fördern oder wie Augmented Reality-Umgebungen das Verständnis von Strukturen bzw. Struktur-Funktions-Zusammenhängen unterstützen. Zusätzlich ist der jeweilige Kontext Teil des TPACK-Modells (Mishra und Koehler 2006), d. h. mögliche fördernde bzw. hemmende Rahmenbedingungen. So ist es bspw. von Bedeutung, wie das Lernumfeld beschaffen ist oder welche Geräte und Support-Möglichkeiten zur Verfügung stehen.

Allerdings fühlen sich angehende und praktizierende Lehrpersonen oft nicht adäquat qualifiziert (Chai et al. 2013). Sie sind also nicht davon überzeugt, dass sie das eben beschriebene Wissen und die Fähigkeiten besitzen, die notwendig wären, digitale Technologien lernförderlich zu nutzen. Diese Wahrnehmung des eigenen Wissensbestands wird auch als akademisches Selbstkonzept einer Person beschrieben (Shavelson et al. 1976).

Das Selbstkonzept stellt einen kognitiven Bereich motivationaler Orientierungen von Lehrpersonen dar (Baumert und Kunter 2006; Paulick et al. 2016). Shavelson et al. (1976) beschreiben das Selbstkonzept als die Selbstwahrnehmung einer Person. Es lassen sich ein nicht-akademisches Selbstkonzept sowie ein akademisches Selbstkonzept unterscheiden. Das akademische Selbstkonzept steht im Mittelpunkt dieses Beitrags. Dieses bezieht sich dabei auf spezielle Inhaltsbereiche (Hattie 1992; Stiensmeier-Pelster und Schöne 2008), bei angehenden Lehrpersonen beispielsweise auf ihr Professionswissen. Paulick et al. (2016) konnten zeigen, dass sich auch im akademischen Selbstkonzept die drei Wissensbereiche des Professionswissens nach Shulman (1986, 1987; Fachwissen, fachdidaktisches Wissen und pädagogisch-psychologisches Wissen, siehe unten) abbilden lassen. Dabei korrelieren die Bereiche des akademischen Selbstkonzepts jeweils am stärksten mit dem korrespondierenden Wissensbereich. Diese Korrelationen liegen im moderaten Bereich.

Der vorliegenden Studie liegt die Annahme eines Mittelwegs zwischen einem transformativen und einem integrativen Modell des TPACK als korrespondierende Wissensbasis für das akademische Selbstkonzept zugrunde, der im Folgenden kurz erläutert werden soll. Die Unterscheidung zwischen integrativem und transformativem Modell stammt ursprünglich aus der Forschung zur Entwicklung des PCK (Gess-Newsome 1999). In der *integrativen* Konzeptualisierung existiert PCK nicht als eigenständiger Wissensbereich, sondern entsteht im Laufe der Entwicklung des Professionswissens aus der Integration von CK und PK. PCK kann also als „Mischung“ von CK und PK beschrieben werden, bei dem die „Zutaten“ immer sichtbar bleiben. Die *transformative* Sichtweise versteht PCK als eigenständige Domäne. PCK ist dabei ein Amalgam aus CK und PK, welches während der Wissensentwicklung entsteht. Die „Zutaten“ CK und PK in diesem Verbund nicht mehr sichtbar. Nach diesem Verständnis ist für jeden fachlichen Kontext und unterschiedliche Lerngruppen ein eigenes PCK erforderlich, weil nicht mehr flexibel auf die anderen Wissensbestände zugegriffen werden kann. Diese beiden Perspektiven stellen also Extreme dar. Der oben genannte Mittelweg versteht die Wissensbereiche jeweils als eigenständig. Die Wissensbereiche stehen dabei nicht unverbunden zueinander, sondern es zeigen sich Zusammenhänge. Diese Struktur ließ sich bereits für das Professionswissen (z. B. Großschedl et al. 2014) als auch für das akademische Selbstkonzept zum Professionswissen zeigen (Paulick et al. 2016).

Motivationale Orientierungen wie das akademische Selbstkonzept beeinflussen, ob angehende Lehrpersonen digitale Technologien später auch tatsächlich einsetzen. Bisher liegen jedoch noch keine validierten Instrumente vor, die die aktuelle Forschung zum akademischen Selbstkonzept und zum technologiebezogenen Professionswissen berücksichtigen, eine Nutzung im deutschsprachigen Raum ermöglichen und eine spezifische Ausschärfung für das Unterrichtsfach Biologie besitzen.

## Fragestellungen und Hypothesen

Ziel des vorgestellten Projektes war es daher, ein Instrument zur Erfassung des akademischen Selbstkonzepts zum technologiebezogenen Professionswissen von angehenden Biologielehrpersonen zu entwickeln und zu validieren.

Bevor die konkreten Forschungsfragen vorgestellt werden, soll eine kurze Einordnung des Validitätsverständnisses in dieser Studie erfolgen. Kane (2001) gibt einen guten Überblick über die Veränderung des Verständnisses von Validität über die Zeit sowie Hinweise, was bei Validierungsstudien zu beachten ist. Einige Aspekte aus seinen Ausführungen sollen hier kurz thematisiert werden. Zunächst ist es wichtig, dass Validität nicht als Merkmal des Instrumentes selbst verstanden wird. Vielmehr steht die Plausibilität der Interpretation im Mittelpunkt, die man mithilfe der mit dem Instrument erhobenen Daten vornehmen will. Die Interpretation dieser Daten sollte darüber hinaus einen sinnvollen Vorteil bieten. Man sollte also grundsätzlich prüfen, was genau man für welche Personengruppe und für welches Einsatzszenario mit welchem Ziel messen will (also in unserem Fall: das akademische Selbstkonzept zum TPACK von angehenden Lehrkräften, um dieses Konstrukt in der wichtigen Professionalisierungsphase des Studiums untersuchen zu können).

Die die Studie leitenden Forschungsfragen und Hypothesen werden im Folgenden nach Validitätsaspekten strukturiert vorgestellt.

### Konstruktvalidität (diskriminante und konvergente Validität)

Zunächst einmal ist es notwendig, die Operationalisierung hinsichtlich der Modellpassung zu überprüfen. Dabei wird der Frage nachgegangen, ob sich die sieben Dimensionen aus dem Modell von Mishra und Koehler (2006) auch empirisch nachweisen lassen (*diskriminante Validität*):

1. Lassen sich die sieben angenommenen Bereiche des TPACK-Selbstkonzepts empirisch trennen?

Aufgrund empirischer Befunde zur Trennbarkeit des akademischen Selbstkonzepts (z. B. Paulick et al. 2016) und des Professionswissens (z. B. Großschedl et al. 2014) wird angenommen, dass sich das sieben-dimensionale Modell im Vergleich zu Modellen mit weniger Dimensionen als das am beste zu den Daten passende erweist.

Weiterhin stellt sich die Frage, ob sich das Instrument ähnlich verhält, wie andere Instrumente, die vergleichbare Konstrukte messen (*konvergente *Validität):

2. Welche Zusammenhänge zeigen sich zwischen dem vorgestellten Instrument und einem bewährten Instrument zur Erfassung des akademischen Selbstkonzepts?

Es wird erwartet, dass die analogen Konstrukte (z. B. CK mit CK) höher miteinander korrelieren als nicht-analoge Konstrukte (z. B. CK mit PK und PCK), und dass die technologiebezogenen Konstrukte höher mit dem analogen nicht-technologiebezogenen Konstrukt korrelieren (z. B. TCK mit CK) als mit nicht analogen nicht-technologiebezogenen Konstrukten (z. B. TCK mit PK und PCK).

### Kriteriumsvalidität (konkurrente Validität)

Neben Fragen zur Konstruktvalidität soll das Instrument auch bezogen auf die Kriteriumsvalidität untersucht werden. Dabei geht es darum, inwiefern das Instrument die Stichprobe erwartungskonform beschreiben kann. Validierungsstudien müssen kritisch betrachtet werden, wenn sie ausschließlich eine Auswahl nicht theoretisch begründeter Variablen berücksichtigen (Kane 2001). Deswegen sollen die ausgewählten Variablen im kurz begründet werden: Sowohl die Phase der Professionalisierung (bzw. der Fortschritt im Professionalisierungsprozess), der Lehramtszugang sowie die studierten Unterrichtsfächer stehen als Indikatoren für die Anzahl von Lerngelegenheiten. Die Annahme ist, dass der Umfang an Lerngelegenheiten positiv mit den Bereichen des Selbstkonzepts in Verbindung steht. Diese Annahme wird durch Forschung zum Professionswissen (z. B. Blömeke et al. 2008; Großschedl et al. 2015; Kleickmann et al. 2013) und zu motivationalen Faktoren (z. B. Andrew und Schwab, 1995; Mahler et al. 2017) unterstützt.

1. Welche Zusammenhänge gibt es zwischen dem TPACK-Selbstkonzept (SK-TPACK) und (a) der Ausbildungsphase, (b) dem Lehramtszugang und (c) der Wahl des Zweitfaches (naturwissenschaftlich vs. nicht-naturwissenschaftlich)?

Es ist zu erwarten, dass die Professionalisierung durch insgesamt mehr Lerngelegenheiten mit einem höheren SK-TPACK einhergeht (Bachelor < Master < Vorbereitungsdienst). Es ist weiterhin davon auszugehen, dass der Lehramtszugang durch mehr Lerngelegenheiten im Fach und der Fachdidaktik mit einem höheren SK-TPACK einhergeht (Sekundarstufe I < Sekundarstufe II). Bezüglich der Wahl des Zweitfaches ist zu erwarten, dass die Probanden mit Biologie und einem weiteren naturwissenschaftlichen Fach durch mehr Lerngelegenheiten in den Naturwissenschaften in den fachbezogenen Skalen (CK, PCK, TCK, TPCK) höhere Werte berichten als Probanden, die Biologie und kein weiteres naturwissenschaftliches Fach als Studienfach haben.

## Methode

### Stichprobe

Zur Validierung, wurde eine Stichprobe von insgesamt 403 angehenden Lehrpersonen aus Schleswig-Holstein und Hessen herangezogen. Die Erhebung fand im Rahmen von Lehrveranstaltungen (im Studium und im Vorbereitungsdienst) als *paper and pencil*-Test sowie als Online-Erhebung statt. 67,5 % der teilnehmenden angehenden Lehrpersonen war weiblich (keine Angabe: 7,2 %). Die Lehrveranstaltungen wurden dabei breit adressiert, um einen guten Querschnitt über die Ausbildungsphasen zu gewährleisten. Die Teilnahme war freiwillig. Die Datenerhebung war vor Beginn der SARS-CoV-2-Pandemie und damit vor dem Beginn der verstärkten Nutzung digitaler Medien im Schul- und Hochschulalltag abgeschlossen. Die Stichprobe bestand aus Proband*innen in unterschiedlichen – aber frühen – Professionalisierungsphasen: 41,44 % befanden sich zum Zeitpunkt der Erhebung im Bachelorstudium, 25,06 % im Masterstudium und 14,88 % im Vorbereitungsdienst (keine Angabe: 18,61). Teilgenommen haben angehende Lehrpersonen für das Lehramt der Sekundarstufe I (22,82 %) sowie für das Lehramt der Sekundarstufe II (67,49 %) (keine Angabe: 9,67 %). 16,63 % der Proband*innen belegten ein zweites naturwissenschaftliches Fach.

### Instrumente

#### Akademisches Selbstkonzept zum technologiebezogenen Professionswissen von angehenden Biologielehrpersonen (SK-TPACK) – MaSter-Bio

Wie im theoretischen Hintergrund bereits erläutert liegt der Instrumentenentwicklung die Annahme eines Mittelwegs zwischen einem transformativen und einem integrativen Modell des TPACK als korrespondierende Wissensbasis für das akademische Selbstkonzept zugrunde.

Dieser Perspektive folgend wurden Items für alle sieben Bereiche des akademischen Selbstkonzepts entwickelt. Den Ausgangspunkt für die Instrumentenentwicklung stellt die Arbeit von Schmidt et al. (2009) dar. Trotz der oben erwähnten Problematik bezüglich der Unterscheidung zwischen Wissen und Selbstkonzept als Erhebungsgegenstand, bietet dieses Instrument eine sehr gute Ausgangslage, weshalb es hier adaptiert und weiterentwickelt wurde. Dieses englischsprachige Instrument bezieht sich auf die sieben technologiebezogenen Bereiche des SK-TPACK. Im Rahmen dieser Arbeit wurde das Instrument zunächst übersetzt und dann sowohl fachspezifisch ausgestaltet als auch durch neu entwickelte Items gestärkt. Die Übersetzung erfolgte durch die beiden Autorinnen. Im Original-Instrument (Schmidt et al. 2009) ist die Fachspezifität schon angelegt. Jedoch bezieht es sich auf verschiedene Fächer („mathematics“, „literacy“, „science“ und „social studies“, S. 147). Zudem werden die Naturwissenschaften unter „sciences“ zusammengefasst. Da in den deutschsprachigen Ländern Biologie meist als separates Fach unterrichtet wird, wurde das Instrument auf die Verwendung im Biologieunterricht angepasst bzw. entsprechend reduziert. Durch die Fokussierung auf ein einziges Fach blieben häufig nur ein-Item-Skalen, die dann durch Items ergänzt wurden. Dabei wurden bspw. in Bezug auf PCK und TPCK analog zu vorhandenen Studien Items in Bezug auf Instruktionsstrategien, Repräsentationsformen sowie Schüler*innenvorstellungen ergänzt (Großschedl et al. 2015; Schmelzing et al. 2013). Durch diese Fokussierung und Erweiterung soll eine Messung für das TPACK-Selbstkonzept mit Bezug auf den Biologieunterricht ermöglicht werden. Das Instrument wurde unabhängig voneinander zwei Studierenden vorgelegt. Diese wurden gebeten, den Fragebogen zu bearbeiten und zu kommentieren und dabei laut zu denken. Dies diente einerseits der Überprüfung der Verständlichkeit der Übersetzung und Ergänzung und andererseits der Abschätzung, wieviel Zeit die Bearbeitung in Anspruch nimmt. Anschließend wurde das Instrument entsprechend der Hinweise der Studierenden überarbeitet. Ferner wurde das Instrument von einer zweiten Arbeitsgruppe, die zum gleichen Thema, aber nicht im gleichen Projekt arbeitet – also entsprechende Expertise hat – kommentiert. Auch diese Anmerkungen wurden zur Optimierung des Instruments genutzt. Die finale Version der Items sowie ein Hinweis zum Ursprung dieser finden sich in Anhang 1.

Sieben Subskalen bilden also die sieben angenommenen Bereiche des akademischen Selbstkonzepts zum technologiebezogenen Professionswissen im Biologieunterricht ab. Drei Subskalen sind folglich nicht technologiebezogen (SK-CK, SK-PCK, SK-PK), vier Subskalen repräsentieren die Bereiche mit Technologiebezug (SK-TK, SK-TCK, SK-TPK, SK-TPCK). Eine fünfstufige Likert-Skala gibt den Proband*innen die Möglichkeit sich bezüglich einer Aussage zu positionieren (z. B. *„Ich lerne technische Dinge schnell.“*; (1) stimme zu, (2) stimme eher zu, (3) neutral, (4) stimme eher nicht zu, (5) stimme nicht zu). Zusätzlich dazu haben die Proband*innen die Möglichkeit keine Angabe zu machen.

#### Akademisches Selbstkonzept zum Professionswissen – BevaKomP

Um die konvergente Validität sicherzustellen, wurde ein Instrument eingesetzt, welches das akademische Selbstkonzept zum Professionswissen im Lehramtsstudium erfasst (BevaKomP, Braun et al. 2008). Dieses Instrument hat keinen Technologiebezug und wurde bereits in zwei Großprojekten eingesetzt (*Information für das Peer-Review entfernt*, z. B. Kleickmann et al. 2014; Paulick et al. 2016; *Information für das Peer-Review entfernt*; z. B. Großschedl et al. 2018). Es enthält drei Subskalen mit jeweils sieben vierstufigen Likert-Items ((1) trifft gar nicht zu, (2) trifft eher nicht zu, (3) trifft eher zu, (4) trifft völlig zu). Die Proband*innen positionieren sich bezüglich einer Aussage (z. B. „Ich kann wichtige Begriffe/Sachverhalte aus diesem Studienbereich wiedergeben.“) jeweils zu den Bereichen (1) Fach Biologie (CK), (2) Fachdidaktik Biologie (PCK) und (3) Bildungs- bzw. Erziehungswissenschaft (PK). Tab. 2 gibt einen Überblick über die Skalen.

**Table 2:** 

Skala	*N*_items_	*M*	*SD*	Cronbach’s α
BSK-CK	3	11,83	2,36	0,85
BSK-PK	7	25,89	4,59	0,87
BSK-PCK	4	10,94	3,31	0,87

### Statistische Auswertung

Als Aspekte der Testgüte werden Reliabilität und Validität herangezogen. Dabei wird auf diskriminante, konvergente und konkurrente Validität eingegangen.

Um Hinweise für die *diskriminante Validität als Aspekt der Konstruktvalidität* zu finden (Forschungsfrage 1), wird im Rahmen einer konfirmatorischen Faktorenanalyse (CFA; MPlus Version 7) überprüft, ob sich die als eigenständig angenommenen SK-TPACK Bereiche auch tatsächlich empirisch trennen lassen. Dazu werden – unter Berücksichtigung theoretischer und empirischer Vorarbeiten (Kleickmann et al. 2014; Mishra und Koehler 2006; Paulick et al. 2016) vier Modelle spezifiziert. *Modell 1* nimmt an, dass das TPACK-Selbstkonzept durch einen einzigen Faktor abgebildet wird, sich also keine Bereiche unterscheiden lassen. *Modell 2* unterscheidet zwei Faktoren: einen technologiebezogenen Faktor (SK-TK, SK-TPK, SK-TCK, SK-TPCK) und einen Faktor ohne Technologiebezug (SK-CK, SK-PK, SK-TPCK). Das vierfaktorielle *Modell 3* nimmt das SK-TK als einzelnen Faktor an und unterscheidet weiterhin einen Faktor ohne Technologiebezug (SK-CK, SK-PK, SK-TPCK) und einen mit Technologiebezug (SK-TPK, SK-TCK, SK-TPCK). *Modell 4* schließlich nimmt alle Bereiche als eigenständig an und ist entsprechend siebenfaktoriell. Um die Passung des Modells mit den Daten einzuschätzen, werden der *Comparative Fit Index* (CFI), der *Tucker-Lewis Index* (TLI), der *Root Mean Square Error of Approximation* (RMSEA) und das *Standardized Root Mean Residual *(SRMR) herangezogen. Ein CFI sowie TLI > 0,95, ein RMSEA < 0,06 sowie ein SRMR < 0,08 weisen auf eine sehr gute Modellpassung hin (Hu und Bentler 1999; Yu 2002). Ein zusätzlicher χ^2^-Differenztest stellt sicher, dass das favorisierte Modell sich auch signifikant von den anderen Modellen unterscheidet.

Schreiber et al. (2006) empfehlen für eine CFA eine Stichprobengröße von mindestens 100 Personen und zusätzlich idealerweise eine Personenanzahl von *N* = 10 pro geschätztem Parameter. Mit *N* = 403 angehenden Lehrpersonen haben wir eine große Stichprobe, die die erste Bedingung voll erfüllt. Im Verhältnis zu den geschätzten Parametern ist die Stichprobe mit kleinen Abstrichen als geeignet zu betrachten.

Die verwendete Software Mplus (Version 7) schätzt fehlende Werte mithilfe des *full information maximum likelihood*-Verfahrens (FIML; Geiser 2010). Im FIML-Verfahren werden die fehlenden Werte nicht imputiert, sondern es werden die notwendigen Parameter (wie bspw. Kovarianzen) geschätzt. Dabei werden nur Variablen berücksichtigt, die für den jeweiligen Fall auch beobachtet wurden (Enders und Bandalos 2001).

Um Informationen über die *konvergente Validität* als weiteren Aspekt der Konstruktvalidität zu generieren (Forschungsfrage 2), wird das akademischen Selbstkonzepts zum Professionswissen mittels BevaKomP (Braun et al. 2008) herangezogen und Korrelationen mit den Skalen des SK-TPACK berechnet.

Um das SK-TPACK Instrument bezüglich der *konkurrenten Validität *als Aspekt der Kriteriumsvalidität zu überprüfen (Forschungsfrage 3), werden (1) die Phase der Professionalisierung (Bachelorstudium vs. Masterstudium vs. Vorbereitungsdienst), (2) Lehramtszugang (Lehrbefähigung für die Sekundarstufe I vs. Lehrbefähigung für die Sekundarstufe II) sowie (3) die studierten Unterrichtsfächer (kein naturwissenschaftliches Zweitfach vs. naturwissenschaftliches Zweitfach) als zusätzliche Variablen berücksichtigt. Zu diesem Zweck werden MIMIC-Modelle (*multiple indicators multiple cause; *Muthén und Muthén 2007) spezifiziert mit den TPACK-Bereichen als latente abhängige Variablen und den aufgeführten Konstrukten als manifeste Prädiktoren.

## Ergebnisse

### Diskriminante Validität

Zur Überprüfung der diskriminanten Validität als Aspekt der Konstruktvalidität wurde die Faktorstruktur mithilfe einer CFA überprüft (Forschungsfrage 1). Die in Tab. 3 dargestellten Modellfitindizes geben Auskunft über die Modellpassung.

**Table 3:** 

Modell	Df	χ^2^	χ^2^/df	CFI	TLI	RMSEA	SRMR
Modell 1 (1-faktoriell)	665	4656,76	7,00	0,50	0,47	0,12	0,135
Modell 2 (2-faktoriell)	664	3776,33	5,69	0,61	0,59	0,11	0,110
Modell 3 (4-faktoriell)	659	2831,66	4,30	0,73	0,71	0,09	0,099
Modell 4 (7-faktoriell)	644	1269,89	1,97	0,92	0,92	0,05	0,047

*CFI* Comparative Fit Index; *TLI* Tucker-Lewis Index; *RMSEA* Root-Mean-Square Error of Approximation; *SRMR* Standardized Root Mean Residual

Modell 4, welches analog zum TPACK-Modell (Mishra und Koehler 2006) sieben eigenständige Bereiche des Selbstkonzepts annimmt, ist unter Berücksichtigung der oben beschriebenen Modellfitindizes zu bevorzugen. Testet man im Anschluss die anderen Modelle gegen das favorisierte Modell mit einem χ^2^-Differenztest, zeigt sich, dass Modell 4 auch signifikant besser auf die Daten passt, als die anderen Modelle (Modell 4 vs. Modell 1: TRd = 3405,071489, ∆df = 21; *p* = < 0,001, Modell 4 vs. Modell 2: TRd = 3754,051582, ∆df = 20; *p* = < 0,001, Modell 4 vs. Modell 3: TRd = 1449,05585, ∆df = 15; *p* = < 0,001). Die Bereiche des akademischen Selbstkonzepts zum technologiebezogenen Professionswissen sind also empirisch trennbar. Der oben beschriebenen Konzeptualisierung folgend, korrelieren die Bereiche des Selbstkonzepts trotz empirischer Trennbarkeit miteinander (s. Tab. 4). Die technologiebezogenen Bereiche korrelieren schwach bis moderat miteinander. Dabei fällt auf, dass das SK-TK bis auf einen schwachen Zusammenhang zum (SK-PK) nur mit den technologiebezogenen Bereichen in einem Zusammenhang steht. Die Bereiche ohne Technologiebezug korrelieren ebenfalls schwach bis moderat miteinander. Darüber hinaus stehen die Bereiche mit und ohne Technologiebezug auch untereinander in Verbindung.

**Table 4:** 

	SK-CK	SK-PCK	SK-PK	SK-TK	SK-TCK	SK-TPCK	SK-TPK
SK-CK	1	–	–	–	–	–	–
SK-PCK	0,38***	1	–	–	–	–	–
SK-PK	0,21***	0,21***	1	–	–	–	–
SK-TK	0,04	0,01	0,07*	1	–	–	–
SK-TCK	0,26***	0,33***	0,19***	0,23***	1	–	–
SK-TPCK	0,27***	0,39***	0,22***	0,22***	0,54***	1	–
SK-TPK	0,10*	0,21***	0,27***	0,27***	0,43***	0,48***	1

***: *p* < 0,001; **: *p* < 0,01, *: *p* < 0,05

### Deskriptive Statistik und Reliabilität der Subskalen

Die Ergebnisse bezüglich deskriptiver Statistik und Reliabilität fasst Tab. 5 zusammen. Die Skalen zeigen eine gute (Cronbach’s α > 0,80) bis sehr gute (Cronbach’s α > 0,90) Reliabilität.

**Table 5:** 

Skala	N_items_	*M*	*SD*	Cronbach’s α
SK-CK	3	11,83	2,36	0,85
SK-PK	7	25,89	4,59	0,87
SK-PCK	4	10,94	3,31	0,87
SK-TK	7	24,48	6,02	0,91
SK-TCK	4	13,10	3,72	0,84
SK-TPK	5	17,47	3,99	0,90
SK-TPCK	8	26,56	6,95	0,94

### Konvergente Validität

Tab. 6 gibt Auskunft über die zur Überprüfung der konvergenten Validität herangezogenen Korrelationen (Forschungsfrage 2).

**Table 6:** 

	SK-CK	SK-PCK	SK-PK	SK-TK	SK-TCK	SK-TPCK	SK-TPK
BSK-CK^a^	0,37***	0,27***	0,09***	−0,06*	0,18***	0,20***	0,03
BSK-PCK^a^	0,26***	0,32***	0,12***	−0,03	0,14***	0,18***	0,04
BSK-PK^a^	0,01	0,08**	0,06*	−0,02	0,01	0,01	0,04

^a^Skalen aus BevaKomP (Braun et al. 2008); ***: *p* < 0,001; **: *p* < 0,01, *: *p* < 0,05

Erwartungskonform korrelieren die BSK-Skalen jeweils signifikant positiv mit den korrespondierenden Skalen des MaSter-Bio. Dabei fällt auf, dass SK-PK mit BSK-PCK (*r* = 0,117***) etwas höher korreliert als mit dem korrespondierenden BSK-PK (*r* = 0,059***). Hypothesenkonform ist, dass sich zwischen dem SK-TK und den BevaKomP-Items höchstens schwache (negative) Korrelationen (*r* = −0,06) finden lassen. Darüber hinaus zeigen sich signifikant positive Zusammenhänge zwischen den Skalen mit einem Fach- bzw. Fach- und Unterrichtsbezug. SK-TPCK korreliert etwas stärker mit BSK-CK (*r* = 0,203***) als mit BSK-PCK (*r* = 0,176***). Auffällig ist, dass SK-TPK keine Korrelation mit dem analogen nicht-technologiebezogenen Konstrukt (BSK-PK) aufweist.

### Kriteriumsvalidität

Zur Überprüfung der konkurrenten Validität als Aspekt der Kriteriumsvalidität werden der Zusammenhang zwischen den SK-TPACK-Skalen und ausgewählten Prädiktoren untersucht (Tab. 7).

**Table 7:** 

Kriterium	Bereich SK-TPACK	B	*SE*	*p*	*β*
Bachelor vs. Master	SK-TK	0,06	0,10	0,522	0,04
	SK-PK	0,30**	0,10	0,003	0,20**
	SK-CK	0,38***	0,10	< 0,001	0,29***
	SK-PCK	0,34**	0,10	0,001	0,22**
	SK-TPK	0,08	0,11	0,438	0,05
	SK-TCK	0,07	0,12	0,538	0,04
	SK-TPCK	0,06	0,10	0,543	0,04
Master vs. Vorbereitungsdienst	SK-TK	−0,03	0,14	0,838	−0,02
	SK-PK	0,26*	0,13	0,046	0,20*
	SK-CK	0,22*	0,10	0,033	0,19*
	SK-PCK	0,19	0,12	0,114	0,14
	SK-TPK	0,13	0,14	0,350	0,08
	SK-TCK	0,10	0,12	0,398	0,07
	SK-TPCK	0,19	0,15	0,182	0,11
Bachelor vs. Vorbereitungsdienst	SK-TK	0,04	0,13	0,773	0,02
	SK-PK	0,63***	0,12	< 0,001	0,37***
	SK-CK	0,65***	0,13	< 0,001	0,40***
	SK-PCK	0,50***	0,11	< 0,001	0,28***
	SK-TPK	0,23	0,14	0,105	0,13
	SK-TCK	0,18	0,13	0,167	0,11
	SK-TPCK	0,22	0,13	0,086	0,12
SekI vs. SekII	SK-TK	0,04	0,10	0,677	0,02
	SK-PK	0,11	0,10	0,266	0,07
	SK-CK	0,13	0,08	0,123	0,08
	SK-PCK	−0,03	0,10	0,787	−0,02
	SK-TPK	0,07	0,11	0,519	0,04
	SK-TCK	−0,12	0,10	0,230	−0,07
	SK-TPCK	−0,11	0,10	0,288	−0,06
Naturwissenschaftliches Zweitfach	SK-TK	−0,11	0,12	0,326	−0,06
	SK-PK	0,11	0,09	0,224	0,06
	SK-CK	0,27***	0,08	0,002	0,16***
	SK-PCK	0,15	0,10	0,103	0,09
	SK-TPK	−0,10	0,11	0,383	−0,05
	SK-TCK	0,16	0,12	0,161	0,09
	SK-TPCK	−0,03	0,12	0,770	−0,02

Zur Codierung: (1) Bachelor = 0, Master = 1; (2) Master = 0, Vorbereitungsdienst = 1; (3) Bachelor = 0, Vorbereitungsdienst = 1; (4) Sek I = 0, Sek II = 1; (5) kein naturwissenschaftliches Zweitfach = 0, naturwissenschaftliches Zweitfach = 1

#### Ausbildungsphase

Bezüglich der Ausbildungsphase wurden Bachelorstudierende (*n* = 167), Masterstudierende (*n* = 101) und Lehrpersonen im Vorbereitungsdienst (*n* = 60) miteinander verglichen. Masterstudierende und Lehrpersonen im Vorbereitungsdienst erreichen im Vergleich zu Bachelorstudierenden höhere Werte bezüglich ihres akademischen Selbstkonzepts im CK, PCK und PK (*p* < 0,05). Bei den technologiebezogenen Bereichen des akademischen Selbstkonzepts gibt es hingegen keine Unterschiede. Vergleicht man Lehrpersonen im Vorbereitungsdienst und Bachelorstudierende findet sich – abgesehen von einem nicht signifikanten Zusammenhang zwischen SK-PCK und der Ausbildungsphase – ein ähnliches Bild. Die standardisierten Beta-Gewichte sind in dieser Gegenüberstellung erwartungskonform größer als im Vergleich von Bachelor- und Masterstudierenden.

#### Lehramtszugang

Entgegen der Hypothese zeigt sich kein Zusammenhang zwischen dem Lehramtszugang (Studierende des Lehramts für die Sekundarstufe I (*n* = 92) und Studierende des Lehramts für die Sekundarstufe II (*n* = 272)) und den Bereichen des SK-TPACK.

#### Naturwissenschaftliches Zweitfach

Einen weiteren Hinweis bezüglich der Kriteriumsvalidität gibt der Vergleich von Proband*innen mit und ohne naturwissenschaftliches Zweitfach. Proband*innen mit einem naturwissenschaftlichen Zweitfach (*n* = 67) erreichen eine höhere Ausprägung des fachwissenschaftlichen Selbstkonzepts.

## Diskussion und Limitationen

Ziel der hier vorgestellten Studie war es, ein reliables Instrument zur Erfassung des akademischen Selbstkonzepts zum TPACK zu entwickeln und zu validieren. Da die Lehrer*innenbildung eine prägende Phase für die Entwicklung professioneller Kompetenz (Kunter et al. 2013a) und damit auch für das akademische Selbstkonzept darstellt, soll das Instrument bei angehenden Lehrpersonen im Studium und im Vorbereitungsdienst eingesetzt werden.

Eines der meistgenutzten Instrumente zur Erfassung des TPACK-Selbstkonzeptes von Schmidt et al. (2009) wurde als Grundlage für MaSter-Bio genutzt und in Hinblick auf folgende Aspekte erweitert und ausdifferenziert: (1) Berücksichtigung der Forschung zum akademischen Selbstkonzept und zum technologiebezogenen Professionswissen, (2) Nutzbarkeit im deutschsprachigen Raum sowie (3) eine spezifische Ausschärfung für den Biologieunterricht. Die Ergebnisse der Validitätsüberprüfung werden im Folgenden diskutiert.

### Überprüfung der diskriminanten Validität

Die konfirmatorische Faktorenanalyse bestätigt die Annahme, dass das sieben-dimensionale Modell die Daten am besten beschreibt (Forschungsfrage 1). Dies deckt sich mit Befunden zum akademischen Selbstkonzept zum Professionswissen (Paulick et al. 2016) sowie zum Professionswissen selbst (Großschedl et al. 2014; Mahler et al. 2017). Somit können die sieben TPACK-Konstrukte empirisch als trennbar dargestellt und entsprechend als separate Skalen behandelt werden, wodurch differenzierte Aussagen möglich werden. Alle sieben Subskalen sind reliabel. Die plausiblen Korrelationen zwischen den Skalen zeigen aber auch, dass die Konstrukte nicht gänzlich unabhängig voneinander sind. Dies gibt zum einen Hinweise auf die Validität, weil sich erwartungskonform gewisse Konstrukte näherstehen als andere (z. B. die technologiebezogenen Konstrukte untereinander). Dass die Konstrukte, die keine Schnittmenge haben (z. B. SK-CK und SK-TK) nicht bzw. nur schwach miteinander korrelieren, unterstützt die Plausibilität der Korrelationen der anderen Konstrukte. Es zeigt sich also, dass je größer die Schnittmenge ist (z. B. TCK und TPCK haben den Technologiebezug sowie den Fachwissensbezug gemeinsam), desto höher ist die Korrelation zwischen den Konstrukten. Auffällig ist, dass das SK-TK mit keinem anderen Konstrukt in Verbindung steht. Inhaltlich kann man daraus schließen, dass dieser Bereich eine Sonderstellung einnimmt. Da er weder mit dem Fach noch mit dem Unterricht in Verbindung steht, spielt er vermutlich für jede Person, die mit digitalen Technologien arbeitet, eine Rolle. Dies ist für die anderen fach- bzw. unterrichtsbezogenen Bereiche nicht der Fall (s. auch die Argumentation zur Schnittmenge). Entsprechend scheint die fehlende Korrelation plausibel. Entsprechend der Ergebnisse kann von ausreichender diskriminanter Validität ausgegangen werden.

### Überprüfung der konvergenten Validität

Die Hypothese zur konvergenten Validität, der zufolge die Konstrukte des MaSter-Bio mit analogen Konstrukten des BevaKomP (Braun et al. 2008) korrelieren, wird durch die Ergebnisse weitestgehend gestützt. Auffällig ist auch hier, dass das SK-TK wenig in Verbindung mit den BevaKomP-Skalen steht, was zur oben beschriebenen Sonderstellung des Konstruktes passt. Die nicht-hypothesenkonform fehlende Korrelation zwischen beiden Skalen des PK-Selbstkonzepts kann ggf. durch die Operationalisierung erklärt werden. Während sich die BevaKomP-Skalen auf das Lehramtsstudium beziehen, sind die MaSter-Bio-Skalen deutlich unterrichtsbezogener (abgesehen von den Selbstkonzepten zu TK, CK und TCK). Offenbar scheint sich dieser Unterschied insbesondere beim akademischen Selbstkonzept zum PK auszuwirken. Die Ergebnisse geben Hinweise auf die konvergente Validität.

### Überprüfung der Kriteriumsvalidität

Die Hypothese, der zufolge sich die Ausprägungen des SK-TPACK nach der Ausbildungsphase unterscheiden lassen, lässt sich ausschließlich für die nicht-technologiebezogenen Konstrukte bestätigen. Auch ein hypothesenkonformer Unterschied zwischen den Lehramtszugängen (Sekundarstufe I vs. Sekundarstufe II) kann in den Daten nicht gefunden werden. Ähnlich verhält es sich beim Vergleich zwischen Proband*innen mit und ohne zweites naturwissenschaftliches Fach. Hier lassen sich hypothesenkonforme Unterschiede lediglich im Selbstkonzept des Fachwissens finden. Allen drei Hypothesen lag die Annahme zugrunde, dass sich die Gruppen jeweils in den Lerngelegenheiten zu den TPACK-Konstrukten unterscheiden. Die nicht-hypothesenkonformen Ergebnisse könnten dadurch erklärt werden, dass eben diese zugrundeliegende Annahme nicht zutrifft. Dies würde bedeuten, dass weder im Studium noch im Vorbereitungsdienst ausreichend Lerngelegenheiten für den didaktisch begründeten Einsatz digitaler Technologien (im Biologieunterricht) zu finden sind. Dies können wir jedoch nur als vorsichtige Annahme formulieren, da inhaltliche Aussagen nicht aus einer Validierungsstudie abgeleitet werden sollten. Wenn diese Vermutung zutrifft, wäre es sinnvoll, das Instrument entweder zusätzlich bei erfahrenen Lehrpersonen, die häufig digitale Technologien im Unterricht nutzen, einzusetzen, bzw. die Studie einige Zeit nach dem Beginn der durch die SARS-CoV-2-Pandemie verursachten vermehrten Nutzung digitaler Medien (durch Lockdown und Distance-Learning) zu wiederholen, um die Kriteriumsvalidität abzusichern. Die Integration einer systematischen Förderung digitaler Basiskompetenzen in der Lehrer*innenbildung erscheint grundsätzlich sinnvoll, sodass bspw. der Vorstoß der *Arbeitsgruppe Digitale Basiskompetenzen *– die einen entsprechenden Orientierungsrahmen entwickelten (Becker et al. 2020) – unterstützt werden kann.

### Limitationen

Die Ergebnisse stützen die Annahme, dass das vorliegende Instrument geeignet ist, das akademische Selbstkonzept zum TPACK angehender Lehrpersonen zu messen und mithilfe der Messung zu sinnvollen Interpretationen zu kommen. Grenzen der Untersuchung zeigen sich allerdings in zwei miteinander in Beziehung stehenden Aspekten der Wahl der Prädiktoren und der Zusammensetzung der Stichprobe. Die Wahl der Prädiktoren für die Kriteriumsvalidität bezog sich vor allem auf systematische Unterschiede im Lehramtsstudium, die wiederum einen unterschiedlichen Umfang an Lerngelegenheiten implizieren. Zwar lag der Auswahl der Kriterien empirische Evidenz zugrunde (Andrew und Schwab 1995; Mahler et al. 2017; Großschedl et al. 2015; Kleickmann et al. 2013), jedoch zeigen sich dahingehend Grenzen dieser Auswahl, als dass sie nicht pauschal in Zusammenhang zu allen Bereichen des akademischen Selbstkonzeptes zum TPACK gesetzt werden können. Dies wird auch in unseren Ergebnissen deutlich. So zeigt sich für den Prädiktor „Zweitfach“ nur ein Zusammenhang zum Selbstkonzept zum Fachwissen, was in Anbetracht der Anzahl an naturwissenschaftlichen Lerngelegenheiten (die sich ja durchaus in manchen Aspekten wie bspw. den fachgemäßen Arbeitsweisen ähneln) plausibel erscheint. Der Studienfortschritt steht insbesondere in Zusammenhang mit den nicht-technologiebezogenen Bereichen. Ein Grund könnte sein, dass der didaktisch aufbereitete Einsatz digitaler Technologien im Biologieunterricht offensichtlich noch nicht systematisch im Studium berücksichtigt wird. Dies kann jedoch nur eine Annahme bleiben, da ein Instrument nicht gleichzeitig einem Validierungsprozess unterliegen kann und für inhaltliche Aussagen herangezogen werden kann. Entsprechend differenzierte Hypothesen und eine zusätzliche Aufnahme weiterer Prädiktoren (bspw. Lerngelegenheiten mit einem Bezug zu digitalen Technologien) sollten für weitere Untersuchungen unbedingt berücksichtigt werden.

Trotz der Stichprobengröße von 403 angehenden Lehrpersonen unterschreiten wir die von Schreiber et al. (2006) empfohlene Personenanzahl pro Parameter etwas, was vor allem an der siebendimensionalen Modellierung liegt. Die grundsätzlich empfohlene Mindestgröße (*N* > 100) ist jedoch mit der vorhandenen Stichprobe voll erfüllt.

Darüber hinaus ist für die Auswahl der Stichprobe anzumerken, dass es sich um eine Gelegenheitsstichprobe handelt. Dennoch halten wir die Stichprobe aufgrund ihrer Größe, sowie der Abdeckung zumindest zweier Bundesländer und mehrerer Ausbildungsphasen für angemessen, um eine haltbare Aussage bezüglich der Reliabilität des Instruments sowie der Validität zu treffen. Der Einsatz des Instruments für weitere Validierungsschritte – die im Folgenden erläutert werden – erscheint dennoch vielversprechend.

Die Erhebung fand vor der SARS-CoV-2-Pandemie und der damit verbundenen Zunahme der Nutzung digitaler Technologien im Unterricht statt. Es kann vermutet werden, dass sich nicht die Qualität des Konstrukts, sondern v. a. seine Ausprägung bei Lehrkräften verändert hat. Dennoch wären weitere Untersuchungen sinnvoll und interessant.

### Implikationen für weitere Validierungsschritte

Das vorliegende Instrument wurde sorgfältig in Hinblick auf unterschiedliche Validitätsaspekte überprüft. Für eine zusätzliche Absicherung bieten sich zusätzliche Untersuchungen an.

Das vorliegende Instrument wurde hinsichtlich seiner Einsetzbarkeit bei angehenden Biologielehrpersonen geprüft. Aufgrund der deutlichen Unterschiede hinsichtlich der Unterrichtspraxis kann das Ergebnis der Validierungsstudie nicht einfach auf praktizierende Lehrpersonen übertragen werden, sodass ein zusätzlicher Validierungsschritt notwendig ist. Bei praktizierenden Lehrpersonen sollten zur Untersuchung der Kriteriumsvalidität andere Variablen wie die Berufserfahrung, Unterstützungsmaßnahmen an den Schulen, vorhandene Medienkonzepte oder vorhandene technische Infrastruktur genutzt werden. Betrachtet man praktizierende Lehrpersonen, ist auch der Aspekt der prädiktiven Validität interessant. Hier ließe sich untersuchen, inwiefern das akademische Selbstkonzept zum TPACK andere Variablen, wie beispielsweise das korrespondierende Wissen TPACK, Lehrpersonenenthusiasmus, Merkmale der Unterrichtsqualität oder Lernerfolg der Schüler*innen vorhersagen kann.

Darüber hinaus wären weitere Analysen bezüglich der empirischen Struktur bzw. Konstruktvalidität wichtig. Mithilfe einer Multigroup CFA (z. B. Scherer et al. 2017) lässt sich überprüfen, ob sich die angenommene Struktur (hier sieben Subskalen des SK-TPACK) auch über unterschiedliche Gruppen (zum Beispiel Lehrpersonen mit vs. ohne Informatik als Fach) abbilden lässt. In der vorliegenden Stichprobe sind interessante Subgruppen oft zu klein, um eine Multigroup CFA angemessen durchführen zu können. Zukünftig wäre so auch ein Vergleich von angehenden und praktizierenden Lehrpersonen möglich. Dabei ist wichtig zu berücksichtigen, dass die vorhandene Stichprobe angehender Lehrpersonen nicht einfach um praktizierende Biologielehrkräfte ergänzt werden kann. Insbesondere aufgrund der Corona-Pandemie kann eine Vergleichbarkeit nicht gewährleistet werden.

Dem Instrument liegt die Annahme zugrunde, dass das TPACK fachspezifisch ausgeprägt ist. In diesem Beitrag wurde das TPACK-Selbstkonzept lediglich für das Fach Biologie beleuchtet. Das Instrument kann jedoch leicht für andere naturwissenschaftliche Fächer adaptiert werden. Es ist zu erwarten, dass Personen, die bspw. Biologie und Chemie studieren oder unterrichten, zwei getrennte Fragebögen jeweils unterschiedlich beantworten würden. Diese Fachspezifität müsste in folgenden Studien empirisch geprüft werden.

### Implikationen für den Einsatz als Messinstrument

Mit dem MaSter-Bio liegt ein reliables Instrument zur validen Erfassung des akademischen Selbstkonzepts zum technologiebezogenen Professionswissen (TPACK) von angehenden Biologielehrpersonen vor. Folgende Hinweise sollten beim Einsatz berücksichtigt werden:

- Zur Beantwortung des Fragebogens sind etwa 20 Minuten zu veranschlagen.
- Bezüglich der Nutzung des Instruments ist zu betonen, dass dieses *explizit nicht* das TPACK-Wissen adressiert. Für Studien zur Prüfung der Lernwirksamkeit von Interventionsmaßnahmen zur Förderung der TPACK-*Wissens*bereiche ist es daher nur bedingt bzw. indirekt nützlich. Sollen aber der Einfluss solcher Interventionen oder ggf. motivationale Faktoren auf die Intention zur Nutzung oder die tatsächliche Nutzung von digitalen Technologien im Unterricht analysiert werden, kann davon ausgegangen werden, dass dafür das Selbstkonzept relevanter ist als das tatsächliche Wissen (Ajzen 1985). Somit eignet sich ein Fragebogen zum Selbstkonzept – also auch der MaSter-Bio für diesen Zweck besser.
- Da alle sieben Subskalen reliabel sind, kann auch eine Auswahl der Subskalen genutzt werden.

## Fazit

Das MaSter-Bio-Instrument bietet die Möglichkeit das akademische Selbstkonzept zum technologiebezogenen Professionswissen angehender Biologielehrpersonen reliabel und valide zu erfassen. Dabei wurde ein fachspezifischer und ganzheitlicher Ansatz gewählt, der sowohl technologiebezogene Bereiche als auch Bereiche ohne Technologiebezug berücksichtigt. Die Autorinnen laden explizit dazu ein, das Instrument zu adaptieren, um es auch zur Erfassung des Selbstkonzepts zum TPACK anderer Fächergruppen zu nutzen. Aufgrund der inhaltlichen Nähe (bspw. bezüglich fachgemäßer Arbeitsweisen) lässt sich das Instrument ohne großen Aufwand gut auf die anderen naturwissenschaftlichen Fächer anpassen.

## Anhang

### MaSter-Bio

Bitte geben Sie für die Fragen an, inwiefern Sie (nicht) zustimmen:

- ❑ Stimme zu
- ❑ Stimme eher zu
- ❑ neutral
- ❑ Stimme eher nicht zu
- ❑ Stimme nicht zu
- ❑ Keine Angabe

Wenn Sie keine Einschätzung vornehmen können, kreuzen Sie bitte „Keine Angabe“ an.

Technologie ist ein weit gefasster Begriff, der eine Menge verschiedener Dinge bedeuten kann. Für die Zwecke dieses Fragebogens bezieht sich „Technologie“ auf digitale Werkzeuge (Medien). Das beinhaltet bspw. Computer, Laptops, Tablets, Handys, interaktive Whiteboards, Softwareprogramme, etc.

**Table:**

Name	Item
TK01	Ich kann technische Probleme selbst lösen.
TK02	Ich lerne technische Dinge schnell.
TK03	Ich halte mit wichtigen neuen Technologien Schritt.
TK04	Ich spiele häufig mit Technik.
TK05	Ich kenne viele verschiedene Technologien.
TK06	Ich habe die technischen Fähigkeiten, die ich benötige, um Technologien zu nutzen.
TK07	Ich habe ausreichend Gelegenheiten, mit unterschiedlichen Technologien zu arbeiten.
CK01	Ich verfüge über ausreichendes biologisches Wissen.
CK02	Ich kann biologische Denkwege nutzen.
CK03	Ich habe verschiedene Möglichkeiten und Strategien mein Verständnis für Biologie zu entwickeln.
PK01	Ich weiß, wie ich Schüler*innenleistungen im Klassenzimmer beurteilen kann.
PK02	Ich kann meinen Unterricht darauf anpassen, was die Schüler*innen gerade verstehen oder nicht verstehen.
PK03	Ich kann meinen Unterrichtsstil an verschiedene Lerner*innen anpassen.
PK04	Ich kann den Lernprozess von Schülern*innen auf unterschiedliche Arten beurteilen.
PK05	Ich kann ein breites Spektrum an Unterrichtsansätzen im Schulunterricht anwenden (bspw. kooperatives Lernen, direkte Instruktion, forschendes Lernen, problemorientiertes Lernen, Projektunterricht etc.).
PK06	Ich kenne gängige Schüler*innen- und Alltagsvorstellungen.
PK07	Ich weiß, wie Unterrichtsführung (*Classroom Management*) organisiert und erhalten wird.
PCK01	Ich kann in Biologie effektive Lehransätze auswählen, um das Denken und Lernen der Schüler*innen anzuleiten.
PCK02	Ich kann geeignete Instruktionsstrategien nutzen, um biologische Inhalte zu vermitteln.^a^
PCK03	Ich kann unterschiedliche Repräsentationsformen begründet auswählen, um biologische Inhalte zu vermitteln.^a^
PCK 04	Ich kenne typische Schüler*innenvorstellungen zu verschiedenen biologischen Themenbereichen und kann diese in der Unterrichtsplanung berücksichtigen.^a^
TCK01	Ich kenne Technologien, die ich anwenden kann, um Biologie zu verstehen und zu betreiben.
TCK02	Ich kenne Anwendungen und Programme, die biologische Sachverhalte behandeln.^a^
TCK03	Ich kann Anwendungen und Programme nutzen, um biologische Inhalte zu verstehen.^a^
TCK04	Ich kann Technologien nutzen, um Daten aus biologischen Versuchen zu dokumentieren und zu analysieren.^a^
TPK01	Ich kann Technologien für den Unterricht auswählen, um Unterrichtsansätze zu optimieren.
TPK02	Ich kann Technologien für den Unterricht auswählen, die das Lernen von Schülern*innen verbessern.
TPK03	Ich kann kritisch abwägen, wie ich Technologien in meinem Unterricht nutzen kann.
TPK04	Ich kann die Technologien auf unterschiedliche Lehraktivitäten anwenden.
TPK05	Ich kann Technologien im Zusammenhang mit unterschiedlichen Instruktionsstrategien nutzen.^a^
TPCK01	Ich kann Unterricht halten, der biologische Inhalte, Technologien und Unterrichtsansätze angemessen kombiniert.
TPCK02	Ich kann Technologien für meinen Unterricht im Fach Biologie auswählen, die das, was ich lehre, wie ich lehre und was Schüler*innen lernen, verbessern.
TPCK03	Ich kann in meinem Unterricht im Fach Biologie Instruktionsstrategien nutzen, die Inhalt, Technologien und Unterrichtsansätze kombinieren.
TPCK04	Ich kann die Führung übernehmen, anderen zu helfen, die Nutzung von Inhalten, Technologien und Unterrichtsansätze zu kombinieren.
TPCK05	Ich kann Technologien auswählen, die die Vermittlung und Erarbeitung biologischer Inhalte in meinem Unterricht verbessern.
TPCK06	Ich kann Lerngelegenheiten für das Lernen von Biologie mit Technologien schaffen.^a^
TPCK07	Ich kann Lernaktivitäten für das Lernen von Biologie mit Technologien gestalten.^a^
TPCK08	Ich kann Aufgaben für das Lernen von Biologie mit Technologien erstellen.^a^

^a^Bei diesen Items handelt es sich um Neuentwicklungen. Die anderen Items wurden nach Schmidt et al. (2009) übersetzt und ggf. für das Fach Biologie ausgeschärft
